# The double‑edged sword of calling: mediating nursing practice environment and missed care – a cross‑sectional study

**DOI:** 10.3389/fmed.2026.1847280

**Published:** 2026-06-10

**Authors:** Qian Zhao, Guiyue Ma, Nan Mo, Xueqing Zhang, Guiling Miao

**Affiliations:** 1 China Hospital Reform and Development Research Institute of Nanjing University, Nanjing Drum Tower Hospital, Nanjing, China; 2 Department of Respiratory and Critical Care Medicine, Nanjing Drum Tower Hospital, the Affiliated Hospital of Nanjing University Medical School, Nanjing, China; 3 School of Nursing, Anhui University of Chinese Medicine, Hefei, China; 4 Department of Nursing, Second Affiliated Hospital of Anhui Medical University, Hefei, China; 5 Department of Nursing, The First People’s Hospital of Changde City, the Affiliated Hospital of Xiangya School of Medicine, Central South University, Changde, China

**Keywords:** career calling, cross-sectional study, mediation effect, missed nursing care, nursing work environment

## Abstract

**Aim and objectives:**

To investigate the status of missed nursing care and examine the mediating role of calling between nursing work environment and missed nursing care.

**Background:**

Missed nursing care (MNC) critically affects patient safety and care quality and is closely linked to the nursing work environment. However, the role of career calling—a key psychological resource for nurses—in this process remains unclear.

**Design:**

Cross-sectional study.

**Methods:**

We recruited 535 clinical nurses from a tertiary hospital in Nanjing, China, using convenience sampling. Participants completed the Nursing Work Environment Scale, Career Calling Scale, and the Missed Nursing Care Scale. Data were analyzed using descriptive statistics, Pearson’s correlation, and the PROCESS macro in SPSS 26.0. Statistical significance was set at *p* < 0.05.

**Results:**

The mean score for missed nursing care was 34.75 (S*D* = 12.15), indicating a moderate level. Mediation analysis revealed that nursing work environment significantly and negatively predicted career calling (*β* = −0.352, *p* < 0.001), whereas career calling significantly and positively predicted missed nursing care (*β* = 0.138, *p* = 0.034). Furthermore, career calling partially mediated the relationship between nursing work environment and missed nursing care, accounting for 18.1% of the total effect.

**Conclusion:**

Career calling partially mediates the link between the nursing work environment and MNC: a poor environment strengthens calling, which in turn is linked to more MNC, revealing a paradoxical effect.

**Relevance to clinical practice:**

To reduce MNC, nurse managers should improve the practice environment—particularly nurse–physician collaboration and staffing—while fostering a balanced sense of career calling to prevent over-commitment, which is critical for patient safety and care quality.

**Patient or public contribution:**

No patient or public contribution was involved in this study, as the research focused on clinical nurses as the study population.

## Highlights

- This study reveals a potential “double-edged sword” effect of calling in nursing: a poorer practice environment is associated with a stronger sense of calling, which in turn is linked to higher levels of missed care, suggesting that calling may become a risk factor in adverse work contexts.
- It provides dual intervention targets for reducing missed nursing care: improving the practice environment (accounting for 81.9% of the total effect) and guiding calling in a healthy direction to prevent overcommitment.
- The findings offer empirical evidence from clinical nurses in China that can inform nursing management strategies globally, particularly regarding the interplay between practice environment, psychological resources, and care quality outcomes.

## Introduction

The nursing profession serves as the cornerstone of the global healthcare system; however, it currently faces significant challenges including high turnover rates and widespread job dissatisfaction. A critical issue is “missed nursing care” (MNC), which Kalisch defined in 2006 as the omission or delay of required patient care activities, reflecting failures in delivering essential diagnostic, therapeutic, and nursing interventions (1, 2). Empirical evidence indicates that 40–98% of nurses experience episodes of care omission (3–5). MNC can compromise patient safety and contribute to adverse outcomes, including medication errors, falls, hospital-acquired infections, pressure injuries, prolonged hospitalization, decreased patient satisfaction, delayed discharge, and increased mortality (6–8, 41). Furthermore, MNC can adversely affect healthcare systems by exacerbating workplace conflicts, deteriorating work environments, intensifying nurse workload pressures, lowering job satisfaction, and increasing turnover intention among nursing staff (9). Therefore, examining the determinants of MNC and implementing targeted interventions are critical priorities for improving healthcare quality.

The nursing work environment forms a systematic foundation for nursing practice. It encompasses both “hard” environmental features, including hospital infrastructure, compensation, and staffing adequacy (8, 10, 11), and “soft” features, such as nursing leadership, interdisciplinary relationships, and perceived workplace culture (12, 13). Existing research has examined the association between the nursing work environment and MNC. Several studies have indicated that missed care primarily arises from constrained work environments, including insufficient staffing, increased patient care demands, resource limitations, time constraints, and lack of peer support (9). Other studies suggest that hospital setting, unit specialization, shift duration, role clarity, staffing levels, and teamwork further influence MNC (14, 15). An optimal nursing work environment enhances outcomes and facilitates the delivery of high-quality patient care (16). Further investigation is essential to validate the link between the nursing work environment and missed care and to analyze the mediating roles of additional variables in MNC.

The concept of nurses’ career calling as a universally accepted definition has yet to be established. Dobrow et al. (17) proposed a seminal definition that has gained considerable recognition, characterizing career calling as a profound passion for one’s profession, strong professional identity, and persistent goal orientation, all underpinned by a commitment to excellence (17). Empirical evidence demonstrates that career calling correlates significantly with numerous positive outcomes at both the individual and organizational levels. Specifically, nurses with stronger career calling exhibit greater work engagement, enhanced professional identity, and consequently superior job performance and satisfaction, while simultaneously demonstrating lower turnover intentions and actual turnover rates (18, 19). Furthermore, individuals with pronounced career calling typically possess greater career resilience, adaptability, and self-efficacy (18, 43). These competencies collectively enhance the capacity to navigate demanding clinical environments. The nursing work environment may also indirectly influence nurses’ career calling; however, few studies have investigated this indirect effect.

**Figure 1:**
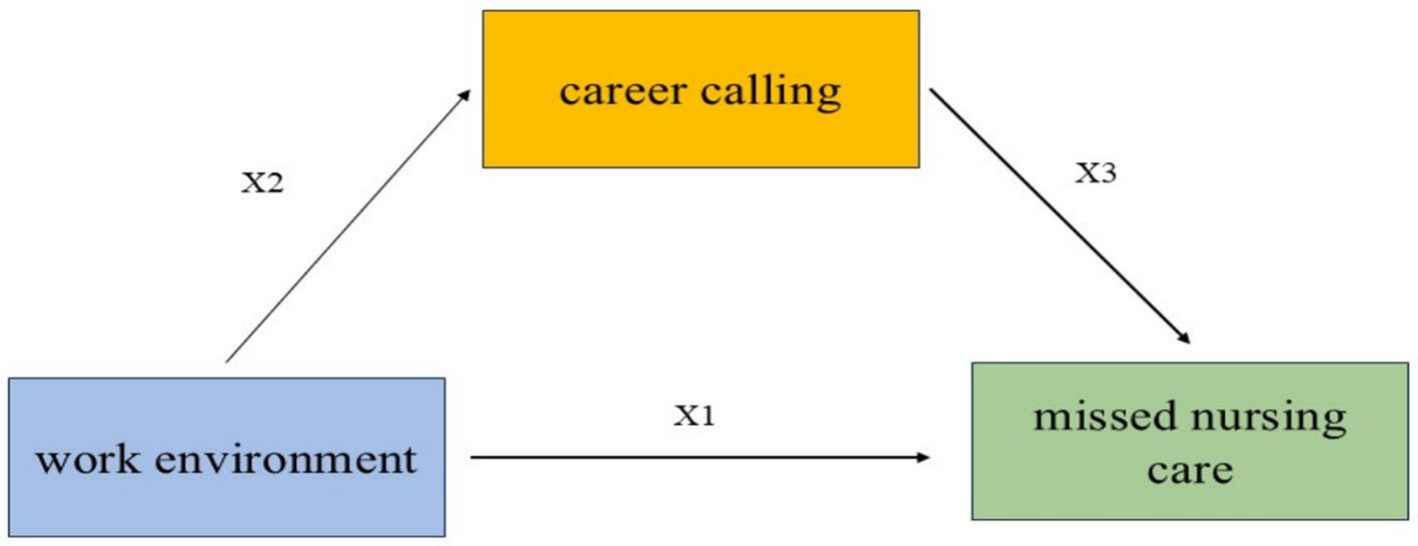
Theoretical model and hypotheses.

Social Cognitive Theory (SCT), developed by Bandura (44), posits that human behavior results from dynamic interactions between environmental, personal, and behavioral factors. This theoretical framework informs our examination of how nurses’ work environment (independent variable) influences MNC (dependent variable), with career calling potentially serving as a mediating mechanism between the two. A supportive practice environment may simultaneously enhance career calling while reducing MNC (20). Consequently, we hypothesized that career calling mediates the relationship between nursing work environments and MNC (Figure 1). This study had two specific objectives: (1) to assess the prevalence of MNC and (2) to examine the mediating role of career calling in the work environment-care omission relationship.

## Methods

### Study design

A cross-sectional study was conducted in a tertiary hospital located in Nangjing, Jiangsu Province, China, from April to September 2025, and this article was reported in accordance with the STROBE guidelines (42).

### Setting and sample

Nurses were recruited from a tertiary Grade A hospital in Nanjing between April and September 2025 Using a convenience sampling method. The inclusion criteria were as follows: (1) possession of a valid nursing practice certificate with completed registration, (2) at least six months of clinical nursing experience, and (3) voluntary participation. The exclusion criteria were as follows: (1) working in non-clinical positions and (2) being on sick or maternity leave during the data collection period.

The sample size was determined using the G*Power (3.1.9.2) program based on the small effect size (*d* = 0.02) recommended by Cohen for multiple regression analysis (21). The sample size (n) was determined to be 395 using the G*Power (3.1.9.2) program (*d* = 0.02, alpha = 0.05, test powe*r* = 80%). Considering the potential dropout rate of 10% and incorrect responses, at least 435 nurses were required. The final sample size was 550, which was adequate according to the hypothesized calculation.

### Measures

#### General information questionnaire

A general information questionnaire was designed by the research team to collect the demographic and career characteristics of the participating nurses (22). Demographic characteristics included age, gender, educational level, and marital status. Career characteristics included professional title, position, years of clinical experience, annual income, overtime hours per week, clinical department, one-way commuting time, job satisfaction, and turnover intention.

#### The nursing work environment scale (NWES)

The Chinese version of the Nursing Work Environment Scale (NWES), developed by Shao et al. (12), was used in this study. The scale comprises 26 items across seven dimensions: professional autonomy, career development, nurse–physician relationship, leadership and management, foundational resources, staffing adequacy, and recognition climate. Items are rated on a 6-point Likert scale ranging from 1 (“strongly disagree”) to 6 (“strongly agree”), yielding a total possible score ranging from 26 to 156. Higher scores indicate a more favorable nursing work environment. In the present study, the scale demonstrated excellent internal consistency, with a Cronbach’s alpha coefficient of 0.967.

#### The MISSCARE scale

The MISSCARE Scale was originally developed by Kalisch et al. (23) in 2009 and was subsequently translated into Chinese and culturally adapted by Sifei in 2019 (24). This study comprises two independent sections: Part A assesses the frequency of MNC, and Part B examines the reasons for such omissions. In the present study, only Part A was administered to measure MNC. This section consists of 24 items, each rated on a 5-point Likert scale ranging from 0 (“never missed”) to 4 (“always missed”), yielding a total possible score of 0 to 96. Higher scores indicate more frequent MNC. In the present study, Part A demonstrated excellent internal consistency with a Cronbach’s alpha coefficient of 0.967.

#### Career calling questionnaire of 12 (calling Scale-12)

Dobrow et al. (17) developed the 12-item Calling Scale in 2011. The scale comprises 12 items, each rated on a 5-point Likert scale. Scores ranged from 1 (“completely disagree”) to 5 (“completely agree”), yielding a total possible score of 12 to 60. Higher scores reflect a stronger sense of calling, characterized by greater intrinsic work enthusiasm and personal identification with one’s profession. In the present study, the scale demonstrated excellent internal consistency, with a Cronbach’s alpha coefficient of 0.954.

#### Date collection

Data were collected from clinical nurses using the online survey platform, Wenjuanxing (https://www.wjx.cn/) (25). This web-based tool allows broad, geographically unrestricted access. The questionnaire, informed consent form, and completion instructions were uploaded to the platform, and an electronic version with a corresponding link and QR code was generated for distribution. The survey comprised the following sections: informed consent, completion instructions, guidelines, core questionnaire items, and a data confidentiality statement.

Prior to questionnaire distribution, the administrators responsible for survey coordination received training via telephone and WeChat. The investigators distributed the questionnaire link to the head nurses’ WeChat groups at the hospital. After providing informed consent, the head nurses forwarded the link to eligible nurses for completion. Nurses accessed the survey by clicking the link or scanning the QR code and completed it independently without supervision.

To ensure data quality, each IP address was restricted to one submission, respondents were required to complete all items, and questionnaires were excluded if all responses were identical or if the completion time was less than 300 s. By July 2025, 550 nurses had completed the survey, meeting the predetermined sample size requirements. Consequently, the online questionnaire link was deactivated.

#### Ethical approval

All procedures were performed in accordance with the Declaration of Helsinki, and the protocol was reviewed and approved by the Ethics Committee of Scientific Research of Nanjing Drum Tower Hospital, the Affiliated Hospital of Nanjing University Medical School (No. 2025–0111-02). Moreover, the responses were kept confidential and anonymous.

#### Statistical analysis

All analyses were performed using IBM SPSS version 26.0, and PROCESS macro version 4.2. We screened the data for respondent misconduct (e.g., extreme or multiple responses), and the screening was good, and all responses were considered for analysis. Descriptive statistics were calculated based on the characteristics of the data distribution. Continuous variables are presented as medians with interquartile ranges (IQR), while categorical variables are presented as frequency and percentage. Pearson’s coefficient was used to correlate normally distributed quantitative variables. Path analysis was conducted using PROCESS macro-model 4 to examine whether the effect of the nursing work environment (X) on nursing care missed by the nursing staff (Y) was mediated by career calling (M). Initially, we controlled for participant characteristics because of their probable relationship with the dependent variable. The analysis revealed a non-significant association; thus, the model was estimated using the main study variables. Bootstrapping (5,000 samples) with a 95% confidence interval was used to determine the statistical significance of the indirect mediation effect on missed care (26). The indirect effect was considered statistically significant if the 95% confidence interval (CI) did not include zero. To assess potential common method bias, Harman’s single-factor test was performed. An unrotated exploratory factor analysis on all self-report items revealed that the first factor explained 38.6% of the total variance, which is below the commonly accepted threshold of 40% (27). This suggests that common method bias did not substantially influence the results.

## Results

### Participant characteristics

A total of 550 nurses participated and answered the questionnaires; 535 were valid, with an effective response rate of 97.3%. Fifteen (2.7%) questionnaires were invalid (with an outlier or the same option selected for each item). The data showed that 16 (2.99%) males and 519 females (97.01%) participated in the study; 91.40% of them had a bachelor’s degree and the mean age (SD) was 34.48 (7.18) years.Tables 1, 2 show the sociodemographic characteristics of the sample population and the results of the univariate model, respectively. In the univariate analysis, education, annual income, overtime hours per week, turnover intention, one-way commuting time, and job satisfaction were associated with MNC. (Table 2).

**Table 1:** Sociodemographic characteristics in nurses (*N* = 535).

Items	*N*	Percentage (%)
Gender		
Male	16	2.99
Female	519	97.01
Age	34.51 ± 7.11	
<30	131	24.49
30–39	281	52.53
40–50	105	19.63
>50	18	3.37
Education		
Junior college	29	5.42
Bachelor’s degree	489	91.40
Master degree and above	17	3.18
Marital status		
Single	104	19.44
Married	414	77.38
Divorced	17	3.18
Length of service (in years)		
<2	17	3.18
2 ~ 5	73	13.65
6 ~ 10	117	21.87
11 ~ 20	220	41.13
≥21	108	20.19
Annual income (in ten thousands RMB)		
<5	80	14.95
5 ~ 9	227	42.43
10 ~ 19	206	38.50
>20	22	4.11
Professional title (nursing)		
Nurse	20	3.74
Registered nurse	178	33.27
Senior nurse	254	47.48
Associate chief nurse	80	14.95
Chief nurse	3	0.56
Position		
General nurses	459	85.79
Head nurse	76	14.21
Overtime hours per week (hours)		
0	30	5.61
1–5	351	65.61
6–10	121	22.62
11–15	22	4.11
>16	11	2.06
Turnover intention		
Definitely not leaving	367	68.6
Might leave	12	2.24
Uncertain	88	16.45
Might not leave	61	11.4
Definitely leaving	7	1.31
Departments		
Internal medicine	164	30.65
Surgery	250	46.73
Gynecology and obstetrics	18	3.36
Pediatrics	41	7.66
Intensive care unit (ICU)	15	0.56
Operating room	24	4.49
Else	23	4.30
One-way commuting time (hours)		
<0.5	392	73.27
0.5–1	119	22.24
1–1.5	9	1.68
>1.5	15	2.80
Work satisfaction		
Very satisfied	133	24.9
Satisfied	283	52.9
Neutral	117	21.9
Dissatisfied	1	0.2
Very dissatisfied	1	0.2

**Table 2:** Univariable analyses of sociodemographic characteristics among nurses associated with MNC (*N* = 535).

Items	MNC	Z/H	*P*
	M (P25, P75)		
Gender		−0.640	0.522
Male	32.50 (25.75, 46.25)		
Female	31 (25, 43)		
Age			
<30	29 (24, 42)	0.711	0.871
30–39	31 (25, 43)		
40–50	31 (25, 43)		
>50	33.50 (24.75, 41. 50)		
Education		11.507	0.003**
Junior college	32 (25, 46)		
Bachelor’s degree	30 (24, 42)		
Master degree and above	45 (30.50, 63.50)		
Marital status		0.990	0.610
Single	30.50 (24.25, 44)		
Married	31 (25, 43)		
Divorced	30 (24, 42)		
Length of service (in years)		2.506	0.644
≤2	25 (24, 45.50)		
2 ~ 5	33 (25.50, 44.50)		
6 ~ 10	30 (24, 44.50)		
11 ~ 20	30 (25, 40)		
≥21	32 (24, 43)		
Annual income (in ten thousands RMB)		12.911	0.005**
<5	26 (24, 42.75)		
5 ~ 9	30 (24, 42)		
10 ~ 19	33 (26, 43)		
>20	41 (29.25, 48.50)		
Professional title (nursing)		4.057	0.398
Nurse	25 (24, 46.50)		
Registered nurse	31 (25, 43)		
Senior nurse	30 (24, 42.25)		
Associate chief nurse	32 (25, 43)		
Chief nurse	63 (43.50, 68.50)		
Position		−4.137	0.000**
General nurses	30 (24, 41)		
Head Nurse	37 (29.25, 46)		
Overtime hours per week (hours)		20.148	0.000**
0	25 (24, 32.25)		
1–5	30 (25, 43)		
6–10	35 (25, 45)		
11–15	35 (24.75, 49)		
>16	24 (24, 31)		
Departments		16.513	0.011*
Internal medicine	31 (25, 42)		
Surgery	32 (25, 44.25)		
Gynecology and obstetrics	30 (24.50, 44)		
Pediatrics	30 (24, 29)		
Intensive care unit (ICU)	24.50 (29, 40)		
Operating room	32 (25, 43)		
Else	36 (24, 45)		
One-way commuting time (hours)		3.192	0.363
<0.5	30 (25, 43)		
0.5–1	33 (25, 43)		
1–1.5	32 (25.50, 58)		
>1.5	27 (24, 35)		
Turnover intention		21.303	0.000**
Definitely not leaving	29 (24, 40)		
Might leave	39 (31.25, 57)		
Uncertain	35 (26, 48)		
Might not leave	34 (25, 41.50)		
Definitely leaving	27 (24, 41)		
Work satisfaction		80.037	0.000**
Very satisfied	25 (24, 33)		
Relatively satisfied	32 (25, 41)		
Moderate	41 (29.50, 49.50)		
Relatively dissatisfied	26		
Very unsatisfactory	24		

### Correlation among NWE, CC, MNC

The means, standard deviations, and Pearson’s correlation coefficients for all the study variables are presented in Table 3. Correlation analysis revealed that the total score on the Nursing Work Environment Scale was strongly positively correlated with each of its sub-dimensions (*r* = 0.833–0.918, *p* < 0.01), indicating that the total score effectively captured the content of the individual dimensions. The nursing work environment was significantly negatively correlated with career calling (*r* = −0.651, *p* < 0.01), indicating that a less favorable work environment was associated with a stronger sense of calling among nurses. The nursing work environment was significantly negatively correlated with MNC (*r* = −0.399, *p* < 0.01), indicating that a more favorable work environment was associated with lower levels of missed care. Career calling was significantly positively correlated with MNC (*r* = 0.323, *p* < 0.01), indicating that a stronger sense of calling was associated with higher levels of missed care. This counterintuitive finding warrants further investigation.

**Table 3:** Correlation among NWE, CC and MNC (*N* = 535).

	M ± SD	NWE	X1	X2	X3	X4	X5	X6	X7	CC	MNC
NWE	125.13 ± 18.10	1									
X1	24.55 ± 3.57	0.918**	1								
X2	19.27 ± 3.20	0.891**	0.794**	1							
X3	19.88 ± 3.05	0.889**	0.801**	0.748**	1						
X4	14.87 ± 2.25	0.894**	0.801**	0.762**	0.847**	1					
X5	19.44 ± 2.93	0.876**	0.781**	0.773**	0.742**	0.752**	1				
X6	13.43 ± 2.80	0.833**	0.730**	0.666**	0.653**	0.696**	0.631**	1			
X7	13.68 ± 2.76	0.854**	0.703**	0.716**	0.687**	0.694**	0.707**	0.777**	1		
CC	24.99 ± 9.79	−0.651**	−0.568**	−0.555**	−0.555**	−0.580**	−0.562**	−0.592**	−0.611**	1	
MNC	34.75 ± 12.15	−0.399**	−0.357**	−0.352**	−0.384**	−0.353**	−0.345**	−0.281**	−0.381**	0.323**	1

** means *P* < 0.01; *means *P* < 0.05. NWE, nursing work environment; X1, Career Development; X2, Leadership and Management; X3, Nurse‑Physician Relationship; X4, Perceived Recognition; X5, Professional Autonomy; X6, Basic Support; X7, Adequate Staffing; CCQ, care calling; MNC, missed nursing care.

### Results of the path analysis

**Figure 2:**
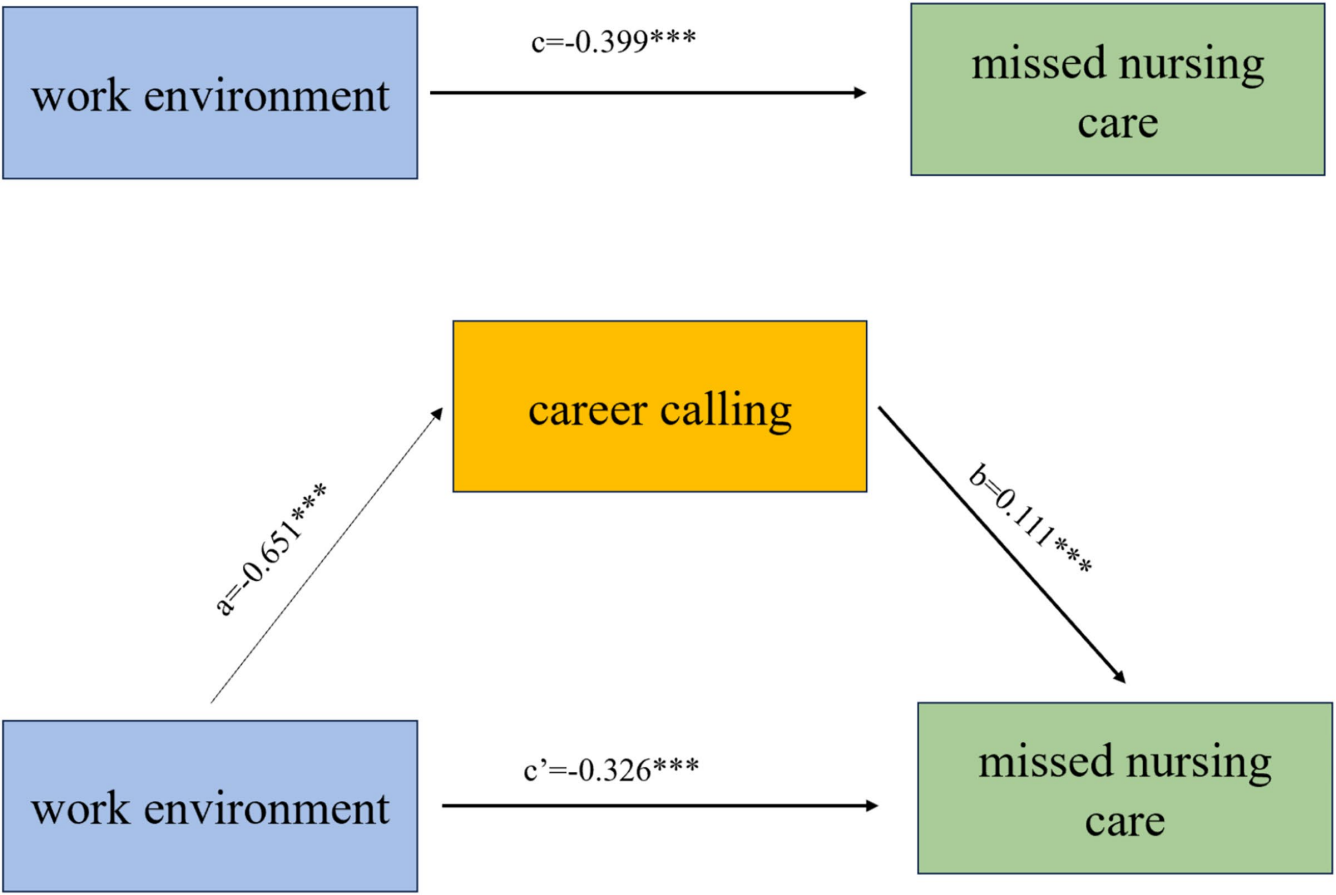
The hypothesized mediation model relating the effect of nursing work environment on missed nursing care through career calling. ***p < 0.001.

In the mediation analysis, c denotes the total effect of the independent variable (X) on the dependent variable (Y), while the product a × b represents the indirect effect (X → M → Y), which captures the mediating effect. To assess the mediating effect, this study employed the product-of-coefficients approach (28–30). This approach, initially proposed by Baron and Kenny (28), tests the significance of the mediating effect by examining whether the confidence interval for a × b contains zero. Following the recommendations of Igartua and Hayes (28), a bias-corrected bootstrap method was employed, resampling 5,000 times to generate 95% confidence intervals and further validate the significance of the mediating effect. All predictor variables were centred prior to the analysis. The results are presented in Tables 4, 5 and Figure 2.

**Table 4:** The mediating effect of nursing work environment, MNC, and career calling questionnaire (*n* = 535).

	MNC		CC		MNC	
	*β*	*t*	*β*	*t*	*β*	*t*
NWE	−0.399	−10.03***	−0.651	−19.80***	−0.326	−6.257***
CC					0.111	2.126*
*R* ^2^	0.159		0.424		0.166	
Adjusted *R*^2^	0.157		0.423		0.163	
*F*	100.65***		392.05***		52.92***	

***means *P* < 0.001; **means *P* < 0.01; *means *P* < 0.05.

**Table 5:** Mediating effect analysis (*n* = 535).

	*β*	SE	*t*	*p*	Bootstrap 95 CI		Mediatio*n* (%)
					LLCI	ULCI	
Total effect (c)	−0.268	0.027	−10.03	0.0000	−0.32	−0.216	
Direct effect (c’)	−0.219	0.035	−6.26	0.0000	−0.288	−0.150	81.9%
Indirect effect (a × b)	−0.049	0.024			−0.097	−0.0021	18.1%

***means *P* < 0.001; **means *P* < 0.01; *means *P* < 0.05.

The total effect of nursing work environment on MNC was significant (*β* = −0.268, *p* < 0.001), accounting for 15.9% of the variance in MNC (R^2^ = 0.159, adjusted R^2^ = 0.157). The nursing work environment significantly and negatively predicted career calling (Path a: *β* = −0.651, *p* < 0.001), while career calling significantly and positively predicted MNC (Path b: *β* = 0.111, *p* = 0.034). The indirect effect was −0.049 (standardized indirect effect = −0.072), with a bias-corrected bootstrap 95% confidence interval of [−0.097, −0.0021], which did not include zero, indicating a significant mediating effect. After controlling for career calling, the direct effect of the nursing work environment on MNC remained significant (*β* = −0.399, *p* < 0.001), indicating that career calling partially mediated this relationship. Finally, to quantify the proportion of the mediating effect relative to the total effect, the ratio (a × b)/c was calculated (29, 30). The results indicated that the indirect effect of career calling accounted for 18.1% of the total effect of the nursing work environment on MNC, suggesting that the mediating pathway plays a substantial role in explaining this relationship. This approach is grounded in the mediation framework originally proposed by Baron and Kenny (28).

## Discussion

This study examined the relationship between nursing work environment and MNC, with career calling as a mediating variable. The results indicated that the nursing work environment was a significant negative predictor of MNC. Career calling partially mediates this relationship, accounting for 18.1% of the total effect. Notably, the mediation pathway revealed a distinctive pattern: the nursing work environment negatively predicted career calling, whereas career calling positively predicted MNC. These findings offer new insights into the mechanisms underlying MNC and provide a theoretical basis for nursing management practices.

In this study, the mean score and SD of MNC was 34.75 (12.15). This is higher than the score of 31.1 (6.4) in the study of 189 nurses by Khajoei et al. (31). This may be due to the different demographic characteristics of the study population. However, the score in this study is slightly lower than 37.11 (14.11) from the study of Jia et al. (32) in hospitals in western China. Possible explanations for this may be the enormous economic disparity between eastern and western China and the different income levels of nurses in these regions. However, the findings of the present study largely echo those of prior research (33), suggesting that inadequate care remains a pervasive issue in clinical nursing practice. The ongoing presence of nursing deficits not only compromises patient safety and quality of care but also exacerbates professional stress and moral distress among healthcare professionals (33). Hence, delving into the root causes and underlying mechanisms of this phenomenon, as well as devising effective intervention strategies, has significant clinical implications.

This study found that the nursing work environment had a significant direct negative predictive effect on MNC (*β* = −0.326, *p* < 0.001); that is, a more favorable work environment was associated with lower levels of MNC. This finding is consistent with those of previous studies (31, 34, 35). According to the Conservation of Resources (COR) theory (36), a favorable work environment provides nurses with sufficient external resources (e.g., staffing and material support) and social resources (e.g., leadership support and teamwork), enabling them to complete nursing tasks more effectively and reduce MNC. Conversely, in poor work environments, nurses face resource shortages and are more likely to selectively omit certain nursing tasks. The presence of this direct effect suggests that improving the nursing work environment is an effective strategy for reducing MNC. Managers should prioritize environmental improvements, particularly in areas such as nurse–physician relationships (*r* = −0.384) and staffing adequacy (*r* = −0.381), which are strongly associated with MNC.

The most salient finding of this study is the mediating role of career calling in the relationship between nursing work environment and MNC. Specifically, the analysis revealed that the work environment significantly and negatively predicted career calling (path a: *β* = −0.651, *p* < 0.001); that is, nurses in less favorable work environments reported a stronger sense of calling. Conversely, career calling significantly and positively predicted MNC (path b: *β* = 0.111, *p* = 0.034). The indirect effect was significant [a × b = −0.072, 95% CI (−0.098, −0.008)], confirming partial mediation, with the mediating effect accounting for 18.1% of the total effect. Although this finding may appear paradoxical—given that career calling is conventionally viewed as a positive psychological asset that inspires responsibility and commitment (37)—it reveals a more complex underlying dynamic. This can be understood from the following perspectives.

First, the “adversity-stimulated” effect of career calling. Career calling is defined as a self-transcendent sense of mission. This profound sense of purpose may be awakened or intensified when individuals encounter challenges or adversity (38). For instance, when nurses encounter unfavorable working conditions, such as understaffing or lack of support, they may experience a heightened sense of calling driven by responsibility toward their patients and the conviction that they must persevere. This phenomenon of “adversity-elicited” calling has also been observed in other professional groups (38). This suggests that career calling is not a fixed psychological trait, but rather a dynamic state that fluctuates in response to environmental changes.

The second is the “double-edged sword” effect of career calling. In recent years, the potential negative consequences of career calling have garnered increasing scholarly attention (39, 40). This study revealed that nurses with a strong sense of career calling had a higher incidence of MNC. This phenomenon may be attributed to the following mechanisms: (1) Excessive workload: nurses with a strong calling tend to assume greater responsibilities and engage in more emotional labor. Even when resources are insufficient, they strive to manage tasks beyond their capacity, leading to an increase in MNC. (2) Heightened self-expectations: Individuals with a strong calling set stricter standards for themselves and may be more vigilant in noticing and reporting deficiencies, whereas those with a weaker calling may overlook or underestimate such omissions. (3) Resource allocation conflict: Nurses with a strong calling may prioritize perceived “high-value” clinical tasks, relegating fundamental nursing care to a secondary position, resulting in selective task omission (8). This finding resonates with recent research on the “dark side” of career calling (40), serving as a reminder to nursing managers to remain vigilant against the risks associated with excessive devotion.

Third, the importance of statistical controls warrants further attention. After adjusting for career calling, the direct effect of the nursing work environment on MNC remained significantly negative (*β* = −0.326), whereas the independent effect of career calling was positive (*β* = 0.111). These findings suggest that career calling assumes an “inhibitory mediating” function between the work environment and MNC: the environment indirectly exacerbates missed care by strengthening career calling, yet the direct inhibitory effect of the environment on missed care outweighs this indirect pathway, thereby neutralizing the adverse consequences of career calling. This observation underscores the importance of distinguishing between direct and indirect effects. Furthermore, it indicates that enhancing the work environment remains the key strategy for reducing MNC while also acknowledging and managing the potential negative effects of career calling in clinical practice.

This study reveals a pathway from calling-overcommitment-missed care. As a psychological resource, career calling is activated in adverse environments and enhances nurses’ sense of responsibility and mission. However, in resource-poor conditions, career calling may become a risk factor for MNC through emotional exhaustion, hypervigilance, and selective task omission. This constitutes the “double-edged sword” effect of career calling. Further analysis suggests that career calling acts as a “suppressive mediator” between the work environment and MNC, and that improving the work environment can effectively reduce MNC. Therefore, career calling is not an unconditional positive resource; in environments with insufficient support, excessive resource depletion may impair care quality. This suggests that nursing management should optimize the work environment, foster a healthy sense of mission, and protect nurses from excessive resource depletion.

## Strengths and limitations

This study delineates the current status of MNC among Chinese clinical nurses and examines their relationship with the nursing work environment and career calling. The findings offer evidence for healthcare administrators to develop targeted interventions for MNC. However, this study had several limitations. First, the single-center convenience sampling at a tertiary hospital in Nanjing may introduce regional bias and limit generalizability. This design also cannot capture cross-institutional moderating effects (e.g., organizational culture, staffing, leadership styles), further restricting external validity. Multi-center, cross-regional studies are therefore needed. Second, the cross-sectional design precludes the establishment of a causal temporal sequence among variables. Although the mediation model is statistically sound and theoretically grounded, the possibility of reverse causality cannot be entirely ruled out. Longitudinal study designs are recommended for future investigations. Third, self-report measures are susceptible to social desirability bias, which may lead to underestimation of MNC and overestimation of career calling. In the future, triangulation using objective indicators and qualitative methods should be conducted. Fourth, the non-significant effects of several demographic and occupational variables do not imply no influence. Their variance may be absorbed by work environment factors or only emerge at higher analytical levels (e.g., cross-level interactions). Multilevel modeling is recommended for future research. Finally, our findings derive from a single Asian healthcare system (China), which may limit their generalizability to non-Asian settings. Although the core mechanisms—calling, overcommitment, missed care—are theoretically universal, cross-cultural differences in professional values, organizational culture, and support systems could moderate these effects. Therefore, future research should replicate our model in diverse geographic contexts (e.g., Europe, North America) to establish cross-national validity and enhance global relevance.

## Conclusion

Among clinical nurses, MNC was relatively common (*M* = 34.75, SD = 12.15). The work environment exerted a direct and significant negative predictive effect on MNC. Furthermore, career calling partially mediated this relationship, accounting for 18.1% of the total effect. Notably, a poor work environment was associated with a stronger sense of calling; however, this heightened calling was linked to increased MNC, revealing the potential “double-edged sword” effect of career calling. These findings suggest that to reduce MNC, managers should foster a healthy and balanced sense of career calling among staff while actively improving the nursing practice environment.

### Relevance to clinical practice

The findings of this study have several implications for clinical nursing management. First, improving the practice environment is the cornerstone strategy for reducing MNC, accounting for 81.9% of the total effect. Managers should prioritise strengthening inter-professional collaboration and ensuring adequate staffing. These two dimensions showed the strongest correlation with MNC (*r* = −0.384 and *r* = −0.381, respectively). Such improvements can be achieved through team-building initiatives, optimized staffing models, and clearly defined communication protocols. Second, the “double-edged sword” effect of career calling warrants careful attention. The mediation path indicates that a poor practice environment intensifies nurses’ sense of mission, which in turn increases MNC, suggesting that a sense of mission may become a risk factor under adverse conditions. Therefore, managers should provide targeted support for nurses with a strong sense of calling to help them balance their professional passion with workload demands, thereby maintaining high-quality care standards. Furthermore, this study highlights that cultivating a positive work atmosphere, rationally allocating resources, and establishing effective communication mechanisms are key to reducing MNC and improving overall care quality.

## Data Availability

The datasets presented in this article are not readily available because the datasets generated and/or analyzed during the current study are not publicly available due to privacy or ethical restrictions but are available from the corresponding author upon reasonable request. Requests to access the datasets should be directed to GMi, 1751655306@qq.com.
